# Development of an HPLC Method with an ODS Column to Determine Low Levels of Aspartame Diastereomers in Aspartame

**DOI:** 10.1371/journal.pone.0152174

**Published:** 2016-03-25

**Authors:** Takashi Ohtsuki, Ryoichiro Nakamura, Satoru Kubo, Akira Otabe, Yoko Oobayashi, Shoko Suzuki, Mika Yoshida, Mitsuya Yoshida, Chiye Tatebe, Kyoko Sato, Hiroshi Akiyama

**Affiliations:** 1 National Institute of Health Sciences, 1-18-1 Kamiyoga, Setagaya-ku, Tokyo, Japan; 2 Ajinomoto Co., Inc., 1-15-1 Kyobashi, Chuo-ku, Tokyo, Japan; 3 Japan Food Research Laboratories, 6-11-10 Nagayama, Tama-shi, Tokyo, Japan; Università di Napoli Federico II, ITALY

## Abstract

α-L-Aspartyl-D-phenylalanine methyl ester (L, D-APM) and α-D-aspartyl-L-phenylalanine methyl ester (D, L-APM) are diastereomers of aspartame (*N*-L-α-Aspartyl-L-phenylalanine-1-methyl ester, L, L-APM). The Joint FAO/WHO Expert Committee on Food Additives has set 0.04 wt% as the maximum permitted level of the sum of L, D-APM and D, L-APM in commercially available L, L-APM. In this study, we developed and validated a simple high-performance liquid chromatography (HPLC) method using an ODS column to determine L, D-APM and D, L-APM in L, L-APM. The limits of detection and quantification, respectively, of L, D-APM and D, L-APM were found to be 0.0012 wt% and 0.004 wt%. This method gave excellent accuracy, repeatability, and reproducibility in a recovery test performed on five different days. Moreover, the method was successfully applied to the determination of these diastereomers in commercial L, L-APM samples. Thus, the developed method is a simple, useful, and practical tool for determining L, D-APM and D, L-APM levels in L, L-APM.

## Introduction

**Fig 1:**
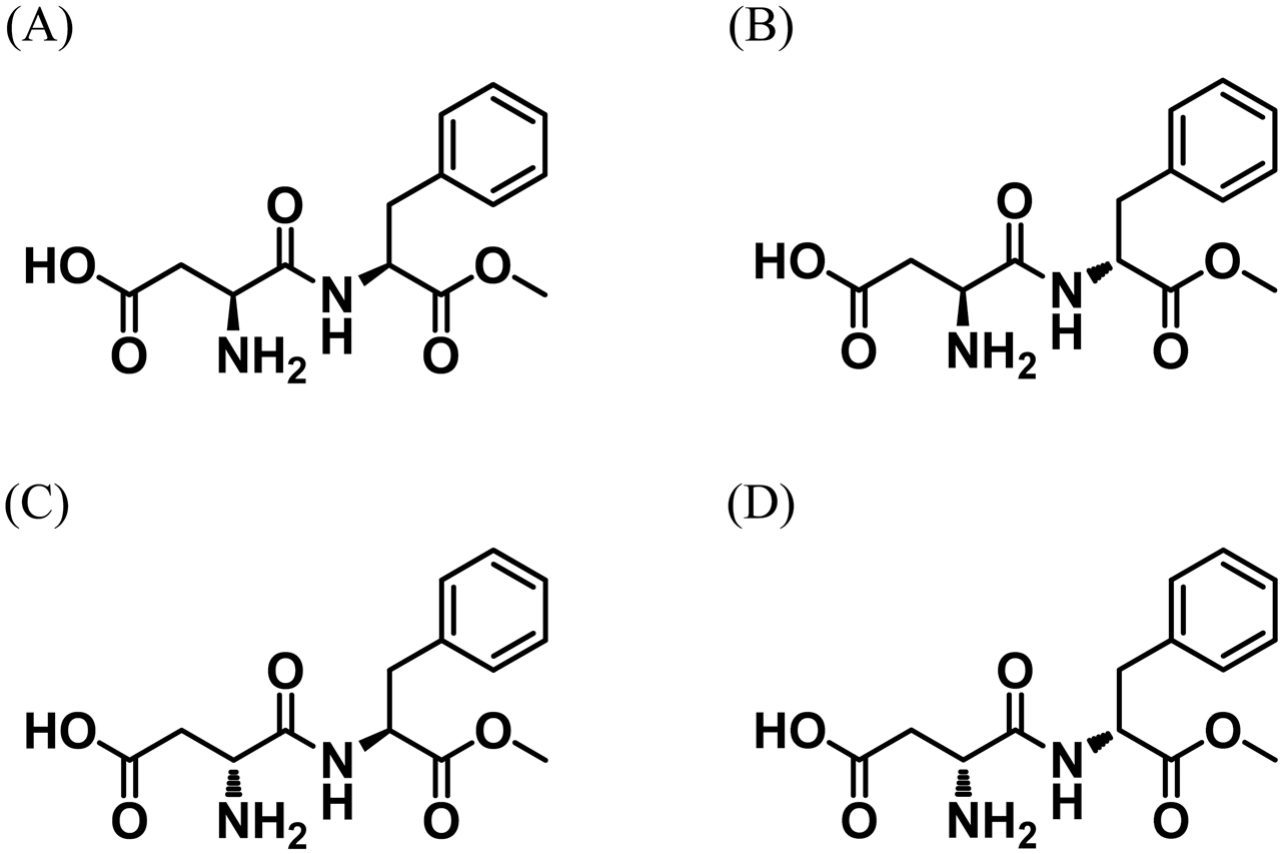
Chemical structures of aspartame (A), α-L-aspartyl-D-phenylalanine methyl ester (L, D-APM) (B), α-D-aspartyl-L-phenylalanine methyl ester (D, L-APM) (C), and α-D-aspartyl-D-phenylalanine methyl ester (D, D-APM) (D).

*N*-L-α-Aspartyl-L-phenylalanine-1-methyl ester (aspartame, L, L-APM; Fig 1A) is an artificial sweetener that is approximately 200 times sweeter than sucrose [1]. L, L-APM is used in more than 125 countries as a food additive in various processed foods such as soft drinks, chewing gum, and tabletop sweetener, as well as in pharmaceuticals [2–4].

Although L, L-APM is relatively stable in the dry state at low temperatures, under certain conditions of pH and temperature in aqueous solution it is well known to decompose into its constituent amino acids, 2-(5-benzyl-3,6-dioxopiperazin-2-yl) acetic acid (DKP), and the isomeric dipeptide-like L-asparaginyl-L-phenylalanine [5–7]. α-L-Aspartyl-D-phenylalanine methyl ester (L, D-APM; Fig 1B), α-D-aspartyl-L-phenylalanine methyl ester (D, L-APM; Fig 1C), and α-D-aspartyl-D-phenylalanine methyl ester (D, D-APM; Fig 1D), the three stereoisomers of L, L-APM, are considered to be byproducts in the synthesis of L, L-APM [8]. Their safety has not yet been assessed, which, as well as the fact that they are known to be bitter or tasteless [9–11], means that it is important to detertmine their presence in L, L-APM. The maximum permitted level for the sum of L, D-APM and D, L-APM in L, L-APM has been limited to 0.04 wt% by the Joint FAO/WHO Expert Committee on Food Additives (JECFA) [12]. The JECFA specifications are particularly important because, as internationally harmonized specifications, they have been adopted in many countries to ensure the safety of food additives.

As specified by JECFA in 1981 [12], the L, D-APM and D, L-APM content in L, L-APM should be determined by amino acid analyzer using a packed column with strong cation exchange resin. However, the specified column is currently not readily available. Other approaches used to determine diastereomers and/or enantiomers of L, L-APM include quantification methods using HPLC with a specific chiral column: possible stationary phases are crown ether [13], α-chymotrypsin [8], or *N*, *S*-dioctyl-D-penicillamine [14]. However, low repeatability and reproducibility between these column lots makes them unsuitable for routine analyses. Recently, the ODS column has been used to determine the main components and impurities in food additives. There has been only one report of the determinaton of L-aspartyl-L-phenylalanine and D-aspartyl-L-phenylalanine, decomposition products of L, L-APM, using HPLC with the ODS column [15], and no studies have established a method using the ODS column to determine L, D-/D, L-APM and L, L-/D, D-APM.

In this study, we developed and validated an HPLC method using an ODS column to determine L, D- and D, L-APM in L, L-APM. We then applied this method to eleven commercial L, L-APM samples.

## Materials and Methods

### Chemicals and reagents

L, L-APM, L, D-APM, D, L-APM, and D, D-APM were kindly provided by Ajinomoto Co., Inc. (Tokyo, Japan) and used as standards for HPLC analysis. Eleven samples of commercially available L, L-APM (samples 1–11) were obtained from domestic and foreign manufacturers. Acetonitrile and methanol were of HPLC grade and purchased from Kanto Chemical Co., Inc. (Tokyo, Japan). Sodium dihydrogen phosphate and disodium hydrogen phosphate were of analytical grade and purchased from Wako Pure Chemical Industries, Ltd. The water used was ultrapure, purified to 18 MΩ·cm using a Milli-Q Gradient A10 (Merck Millipore Corporation, Billerica, MA, USA). All chemicals and samples were weighed using a XS205DU microbalance (Mettler-Toledo International Inc., Columbus, OH, USA).

### Examination of various columns for L, D-APM analysis

To accurately determine the L, D-APM impurities in commercially available L, L-APM using HPLC, full separation of peaks is required. We performed a number of experiments to optimize HPLC conditions such as mobile phase, gradient conditions, detection wavelength, injection volume, flow rate, and temperatures of column oven and auto-injector. To further optimize the ODS column for L, D-APM analysis, we evaluated ODS columns from three different manufacturers (column 1: L-column2 ODS column [4.6 mm I.D. × 250 mm; particle size 5 μm; Chemical Evaluation and Research Institute, Saitama, Japan]; column 2: TSK-gel ODS 80 Ts column [4.6 mm I.D. × 250 mm; particle size 5 μm; Tosoh Co., Kyoto, Japan]; and column 3: Mightysil RP-18 GP [4.6 mm I.D. × 250 mm; particle size 5 μm; Kanto Chemical, Tokyo, Japan]). A test solution, prepared from L, L-APM standard spiked with 0.04 wt% L, D-APM standard, was injected into the HPLC, using each column to compare the chromatographic separation.

### Instruments

Chromatography was performed using a Shimadzu Prominence LC-20AD HPLC system equipped with two LC-20 AD pumps, a SIL-20ACHT auto-injector, a CTO-20AC oven, a DGU-20a degasser, and a SPD-20A UV-visible detector (Shimadzu Corporation, Kyoto, Japan).

### Preparation of standard solutions and sample solutions

Stock solutions of L, D-APM and D, L-APM standards were prepared separately, as follows: 20 mg of the standard was accurately weighed and then placed in a 50 mL volumetric flask. After the standard was completely dissolved in 10% methanol, the volume was adjusted to 50 mL. Stock solutions of L, L-APM and D, D-APM standards were prepared separately, as follows: 5 mg of the standard was accurately weighed and then placed in a 25 mL volumetric flask. After the standard was completely dissolved in 10% methanol, the volume was adjusted to 25 mL.

A calibration curve of L, D-APM was prepared, using six standard solutions (0.2, 0.5, 1.0, 2.0, 5.0, and 10 μg/mL) prepared by diluting the L, D-APM stock solution with 10% methanol.

The sample solutions for HPLC analysis were prepared by placing 100 mg of each sample in a separate 20 mL volumetric flask, which was then filled with 10% methanol. After sonication, these solutions were injected into HPLC system.

### HPLC analysis

The standard and sample solutions (10 μL of each) were analyzed by HPLC. The mobile phase consisted of solvent A (50 mmol/L sodium dihydrogen phosphate:50 mmol/L disodium hydrogen phosphate = 1:1 (v: v)) and B (50 mmol/L sodium dihydrogen phosphate:50 mmol/L disodium hydrogen phosphate:acetonitrile = 4:4:2 (v: v: v)). The gradient conditions were as follows: 0–25 min, 65% solvent B; 25.01–40 min, 100% solvent B at a flow rate of 0.8 mL/min. After each measurement, the column was re-equilibrated for 15 min with 65% solvent B.

Three different columns were investigated. The column temperature was set to 40°C and the detector was operated at an absorbance wavelength of 220 nm. The temperature of the auto-injector was maintained at 5°C during the analysis.

The L, D-APM content in L, L-APM was calculated using the following equation:

$$\text{Content }(\text{wt}\%)=\frac{\text{C}\times \text{V}\times 0.1}{\text{W}}$$

where *C* is the concentration (μg/mL) of L, D-APM in the sample solution, *V* is the volume of the sample solution (20 mL), and *W* is the weight of the sample (mg).

To evaluate the accuracy and precision of the developed method, we performed recovery tests at 0.04 wt%, the maximum level permitted by JECFA for L, D-APM/D, L-APM in L, L-APM, and at 0.01 wt%, over five days (two trials per day). The accuracy of the method was evaluated based on the recovery of L, D-APM in an L, L-APM sample spiked with L, D-APM. The repeatability and reproducibility of the developed method was assessed through repeatability relative standard deviation (RSDr) and reproducibility relative standard deviation (RSD_R_) obtained from one-way analysis of variance (ANOVA) of the recovery rates.

## Results and Discussion

### Examination of various columns for L, D-APM analysis

**Fig 2:**
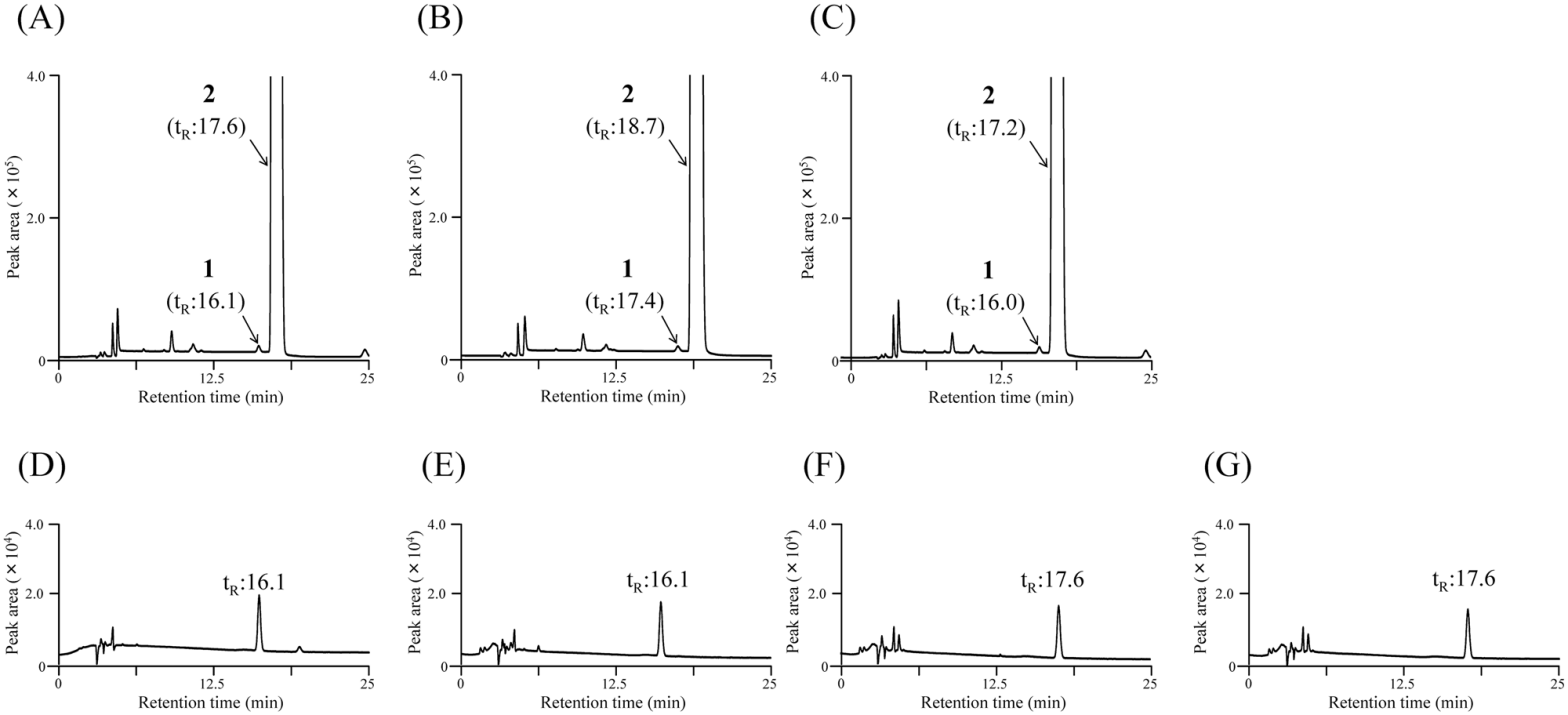
Representative HPLC chromatograms of an L, L-APM standard solution spiked with L, D-APM of 0.04 wt% (A, B, and C), L, D-APM (D), D, L-APM (E), L, L-APM (F), and D, D-APM (G) (all standard solutions; 0.2 μg/mL). Chromatograms A, D, E, F, and G were obtained using the L-column2 ODS column (column 1). Chromatograms B and C were obtained using the TSK-gel ODS 80 Ts column (column 2) and Mightysil RP-18 GP column (column 3), respectively. The peaks labeled 1 and 2 in chromatograms A—C correspond to L, D-APM and L, L-APM, respectively.

We performed a number of experiments to determine appropriate HPLC conditions and found the use of a mixture of sodium dihydrogen phosphate solution, disodium hydrogen phosphate solution, and acetonitrile as the mobile phase allowed full separation of L, D-APM and L, L-APM on the reverse-phase HPLC under the HPLC parameters described in section 2.5 (data not shown). As shown in Fig 2, all tested columns provided sufficient separation between L, D-APM and L, L-APM in the test solution; the resolutions of the separation ranged from 2.00 to 2.20. In addition, all columns provided good peak shapes for L, D-APM with symmetry factors between 1.08 and 1.09.

As shown in Fig 2, columns 1 and 3 allowed relatively short measurement times for L, D-APM and L, L-APM compared with column 2. Moreover, in the case of column 1, the theoretical plate number for L, D-APM, calculated according to the Japanese Pharmacopoeia equation [16], was 21,889 per meter. This number was larger than that of the other columns (column 2: 17,486; column 3: 20,743). Based on these results, we selected column 1 for the subsequent analyses.

As shown in Fig 2, retention times of L,D-APM and L,L-APM were identical to those of their enantiomers (D, L-APM and D, D-APM, respectively) under our HPLC conditions with column 1.

### Linearity and limit of detection and quantification

To determine the linearity and quantification range of the developed method, six L, D-APM standard solutions (0.2, 0.5, 1.0, 2.0, 5.0, and 10 μg/mL) were analyzed and the area of the obtained L, D-APM peak was recorded for each. Linear regression analysis was made by plotting peak area (y) versus L, D-APM concentration (x) in μg/mL. Each measurement was conducted in triplicate.

The mean regression equation of the calibration curve for L,D-APM was found to be y = 6872.87x—99.61, with an excellent correlation coefficient of 1.000 in the concentration range tested, indicating that the method has good linearity. Based on the regression analysis, the limits of detection (LOD) and quantification (LOQ) for L, D-APM, defined as the mass fraction that yields a signal to noise ratio of 3 and 10, respectively, are estimated to be 0.0012 wt% (equivalent to a concentration of L, D-APM of 0.06 μg/mL in test solution) and 0.004 wt% (0.2 μg/mL L, D-APM in test solution), respectively. In addition, we found the LOD and LOQ of D, L-APM to be equal to those of L, D-APM. These levels are much lower than the maximum limit specified by JEFCA for L, D-APM/D, L-APM (0.04 wt%) in L, L-APM. Therefore, the developed method has sufficient sensitivity to determine L, D-APM and D, L-APM in L, L-APM.

### Accuracy and precision

**Fig 3:**
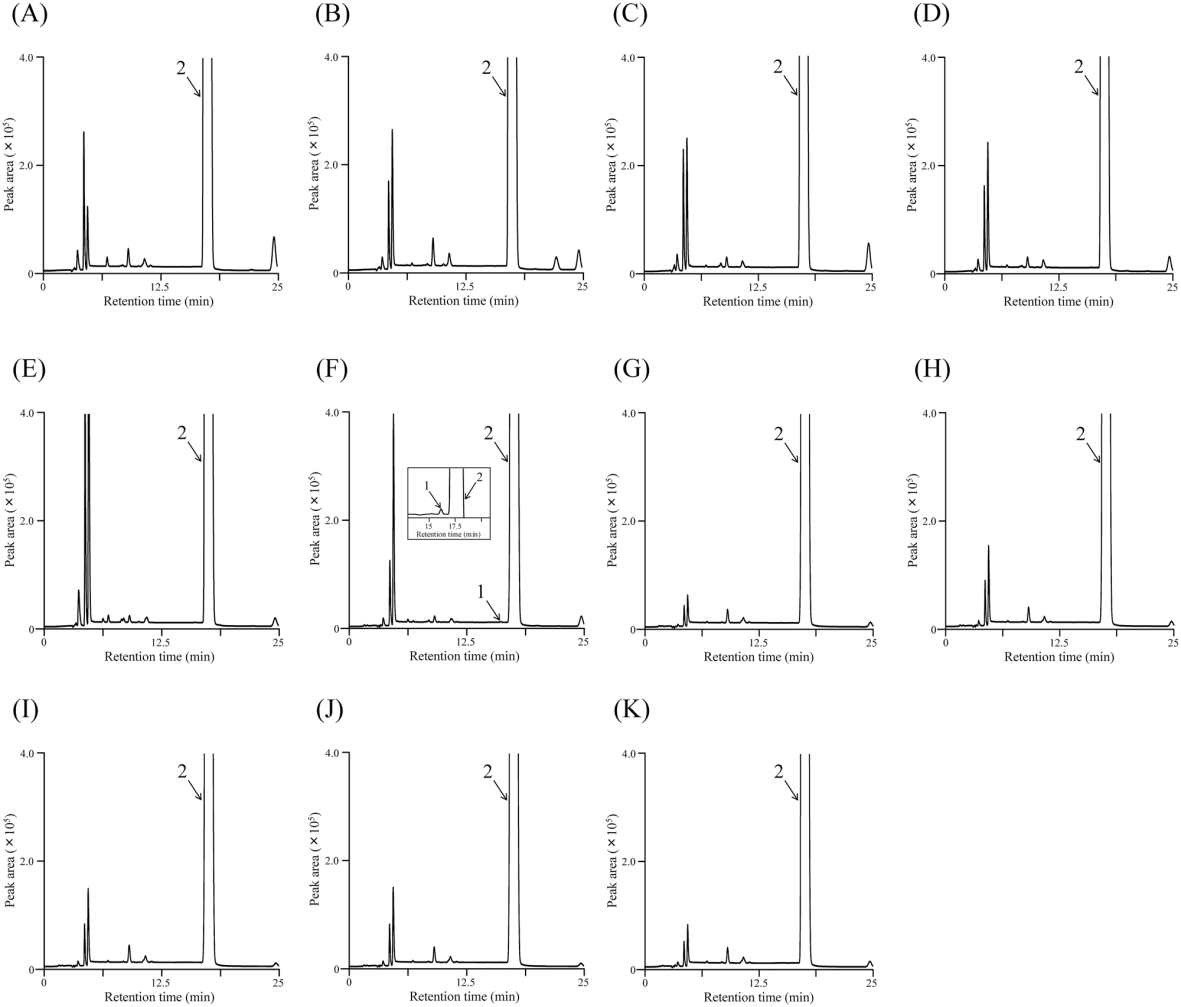
Representative HPLC chromatograms of commercially available L,L-APM samples. The peaks labeled 1 and 2 correspond to L, D-APM and L,L-APM, respectively. These peaks are highlighted in (F).

To evaluate the accuracy and precision of the developed method, we performed recovery tests at 0.04 wt% and 0.01 wt% over five days (two trials per day). Commercial-grade L, L-APM (Sample 11) was used as the blank matrix because its L,D-APM content is less than the LOQ for HPLC analysis, as shown in Fig 3.

As shown in Table 1, the mean values of recovery rates for both concentrations analyzed were greater than 99.1%. In addition, the RSD_r_ and RSD_R_ values were less than 1.0% and 3.5%, respectively, at both concentrations. These results demonstrate that the developed method has satisfactory accuracy and precision and is reliable for determining the content of L, D-APM in L, L-APM.

**Table 1:** Inter-day recoveries, repeatability, and intra-laboratory reproducibility of L, D-APM in L, L-APM.

	Recovery (%)	RSD_r_ (%)	RSD_R_ (%)
0.01 wt%	99.1	1.0	3.5
0.04 wt%	100.7	0.3	0.6

Each recovery value represents the mean of results on five different days (two trials per day). RSD_r_ and RSD_R_ are calculated by one-way analysis of variance of the recovery values obtained on five different days.

### Analysis of commercially available L, L-APM samples

To demonstrate the effectiveness of the developed method, it was applied to eleven commercial L, L-APM samples. Representative chromatograms are shown in Fig 3. The qualitative compositions of L, D-APM and/or D,L-APM and other minor compounds in the commercial L, L-APM samples were different. As shown in Table 2, in all samples except for sample 6, L, D-APM and/or D, L-APM was detected below the LOQ.

**Table 2:** The L, D-APM and/or D, L-APM contents of commercially available L, L-APM samples.

Sample No.	Content (%)	RSD (%)
1	< 0.004	-
2	< 0.004	-
3	< 0.004	-
4	< 0.004	-
5	< 0.004	-
6	0.0044	1.3
7	< 0.004	-
8	< 0.004	-
9	< 0.004	-
10	< 0.004	-
11	< 0.004	-

The L, D-APM and/or D, L-APM of sample No.6 is the mean of three independent measurements.

On the other hand, the mean value for the sum of contents of the L, D-APM and/or D, L-APM in Sample 6 was 0.0044 wt% and the RSD was less than 1.3%. No interference peaks were shown. Accordingly, the method was considered successful in determining the content of L, D-APM/D, L-APM in L, L-APM. In addition, we found the L, D-APM/D, L-APM contents of all samples to be less than the maximum level specified by JECFA (0.04 wt%).

## Conclusion

In this study, we developed and validated an HPLC method to determine low levels of L, D-APM/ D, L-APM in L, L-APM. This is the first report of successful quantification using HPLC with an ODS column. The validation data showed that the developed method provides excellent linearity, accuracy, and precision as well as sensitivity in the assessed concentration range. Furthermore, we proved that the method is applicable to commercial L, L-APM samples. No special HPLC columns or instruments are needed to determine L, D-APM/D, L-APM in this way.

The developed method is simple and versatile as well as useful and practical for the determination of L, D-APM/D, L-APM in L, L-APM; it has the potential to be used as an alternative to conventional method using an amino acid analyzer adapted to JECFA specifications.
